# Breaking all moulds? Racialized romance between white/*yang* women and Chinese men

**DOI:** 10.1080/1070289X.2022.2154013

**Published:** 2022-12-15

**Authors:** Willy Sier

**Affiliations:** Department of Anthropology, Utrecht University

**Keywords:** China, migration, romance, racialization, whiteness

## Abstract

Following increased international migration to China, the number of relationships between foreign women and Chinese men has risen. This article studies how ‘white women’ are racialized in the Chinese context through analysing the meaning of the term *yang*. Based on an analysis of online content about these relationships in English and Chinese, this article demonstrates that different racial frameworks interact when the meaning of race is negotiated in romantic relationships that are not only racialized, but also international and multilingual. Finally, it shows how racial narratives are sometimes reproduced through white/*yang* women’s accounts aimed at negating negative stereotypes about their husbands.

## Introduction

Following China’s integration in the global economy and its increased popularity as a migration destination, the number of marriages between Chinese and foreigners (excluding Chinese citizens and citizens from Hong Kong, Taiwan and Macau) has risen. The gendered pattern of these marriages has also changed. In 1979 only three per cent of the 8460 marriages between Chinese and foreigners were between Chinese men and foreign women, compared with 25 per cent of 49,000 marriages in 2011 (Pan 2014). Media reports show that this number continues to increase (Xiaofei 2017). To date, most research in the field of transnational romance has focused on relationships between Asian women and white, western men (Lapanun 2019; Constable 2003; Kelsky 2001). In this article I turn to the growing number of relationships between white, western women and Chinese men, which is a type of relationship that is generally considered rare and exceptional by scholars, the media and the people in these relationships (Farrer 2008; Farrer and Dale 2014; Nehring and Wang 2016). I analysed 40 blogs on six websites about relationships between white western women and Chinese men living *in* China, often referred to as WWAM relationships – (an acronym for ‘western woman, Asian man’). I study how white-looking women are racialized in China and how these women cope with being confronted with the racial prejudices evoked by being in a racialized relationship.^1^

As scholars have argued, relationships between people ascribed to different racial identities can function as micro sites where racial meanings are negotiated, produced, and reproduced (Twine and Steinbugler 2006). Following earlier studies on racialized relationships in the U.S.A and the UK (Childs 2005; Twine and Steinbugler 2006), recently scholars have analyzed relationships between people ascribed to different racial identities in the Global South, such as in Cuba (Fernandez 2010), Brazil (Osuji 2013), South Africa (Steyn, McEwen, and Tsekwa 2019) and Mexico (Sue 2013). In contrast to the popular notion that relationships that cross racial boundaries are a sign that such boundaries are becoming blurred, these works demonstrate how racialized relationships reveal the pervasiveness of racial divisions and the beliefs that sustain them (Childs 2005), as they evoke scrutiny, stereotyping and sometimes even violence (Perry and Sutton 2008). Scholars describe how couples adjust their behaviour in response to being racialized (Osuji 2013; Steyn, McEwen, and Tsekwa 2019). This behaviour includes limiting their social circles, staying at home, marrying to legitimize their relationship, pretending not to be a couple, and emotionally distancing themselves from – and even denying – racializing discourses (Steyn, McEwen, and Tsekwa 2019). This body of literature focuses on couples who negotiate competing racial projects yet share one language and nationality. Conversely, this article focuses on relationships that are not only racialized, in the sense that one of the first identifying factors of these relationships is that they are made up of people ascribed to different racial categories, but they are also international and multilingual. This is important because it means that in addition to these relationships being sites where partners learn to see from the other person’s racial perspective, as described in the literature on ‘racial literacy’ (Twine and Steinbugler 2006), they are also social spaces where multiple languages and histories of racialization come into interaction with one another.

Scholars have argued for the importance of considering the influence of Euro-American conceptualizations without glancing over local histories and knowledge when studying racializing discourse in non-western contexts (Berg and Wendt 2011; Bonnett 2018). Relatedly, it has been argued that migration entails negotiating multiple racial frameworks (Kim 2008; Joseph 2015; Okura 2021). Keitaro Okura’s recent study (2021) among Chinese students in the U.S.A shows that migration can also lead to people’s discovery of different languages of racialization. Okura’s interlocutors start thinking about themselves as ‘Asian’ in the U. S. A, whereas in China they had considered themselves as ‘Chinese’ or ‘Han’. Similarly, I study how ‘white women’ in China start identifying with the Chinese term *yang*, which is ascribed to them as part of the word *yangxifu*, or *yang-*wife. Literally, this word translates as ‘overseas wife’ and does not explicitly refer to skin colour, yet it is generally used to refer to women who are perceived as being phenotypically white. In this article, I use the term white/*yang* to indicate that I refer to ‘white women’ in the way that this category exists in the Chinese context.

By bringing together debates about racialized relationships and multiple racial languages, I develop three arguments. Firstly, I investigate how ‘white women’ are racialized in the Chinese context, introducing the Chinese term *yang*. I explain why and how this term has come to be associated with whiteness and explore the process of becoming a *yangxifu*. Then, I demonstrate the importance of considering the influence of multiple racial frameworks when researching international and multilingual couples. Finally, I reflect on the way that women write about confronting racism and show that racializing language is sometimes reproduced through anti-racist activism. However, first, I sketch the context in which these relationships should be understood and elaborate on my research methods.

## Marrying into China

The increased migration to China by people who are young, more often female, open to adventure and keen to seek cultural connections and understandings (Camenisch 2019; Farrer 2010; Lehmann and Leonard 2019) has led to a spike in relationships between foreigners and Chinese citizens in China. While scholars have noted that male migrants who are part of this wave become romantically involved with Chinese women, they have paid less attention to relationships between Chinese men and foreign women, asserting that these relationships are relatively rare (Farrer 2008, 2010; 1226; Farrer and Dale 2014; Nehring and Wang 2016; 3; Leonard 2019, 169).

Chinese men’s relationships with white/*yang* women have been explained as a result of China’s rise as an economic powerhouse, which enables Chinese men to use their wealth and status to cross racial divides, a privilege that has long been understood as belonging exclusively to white men (Fernandez 2010, 29). In discussions on China’s dating landscape, relationships between Chinese men and white/*yang* women are often either ignored or presented as very unlikely. Popular blogs describe how white western men are treated as ‘god-like figures’ by Chinese women (Stephanie 2016; Vahle 2020) and live as if they are on a ‘permanent spring break’, while ‘foreign women’ – which refers to white, western women in these writings – have been described as living in ‘a dating wasteland’ (Nilsson 2006) due to their inability to compete with Chinese women over foreign men and their incompatibility with Chinese men. This incompatibility is explained as Chinese men finding women from western countries ‘too dominant’ (Stephanie 2016) and, according to an article in *China Daily*, due to Chinese men being too shy, working too hard, and being afraid of losing face when approaching foreign women (Nilsson 2006). Following from these narratives, the increased number of relationships between Chinese men and white/*yang* women is understood as a testimony to Chinese men’s growing confidence.

Researcher Jiehai Zhang, a sociology professor at the Shanghai Academy of Social Sciences, has investigated white western women’s perceptions of Chinese men and provided an alternative explanation for why relationships between the two groups are uncommon (2009). Zhang’s more than 100 ‘Caucasian’^2^ research participants, who were all living in China, scored Chinese men high on topics such as ‘looking after one’s family’, ‘willing to spend money on women’, and ‘relatively serious about relationships between men and women’. Yet these respondents also identified Chinese men’s weaknesses as being ‘not so gentlemanly’, having a ‘poor physique, not enough exercise’, and ‘no personality, lacking unique opinions’. Moreover, nearly all participants in this study made negative comments about Chinese men’s ‘nasty habits’, such as ‘spitting everywhere’, ‘growing long nails to pick their ears and nose’, and ‘not brushing their teeth well enough’. These comments shed light on how racial stereotypes shape these women’s thinking about their compatibility with Chinese men.

Nevertheless, in recent years it has become increasingly common to see couples made up of Chinese men and white/*yang* women in the streets of China’s big cities. Interest in the *yangxifu*, or the ‘overseas wife’, is also evident from the number of online discussions in which this term appears. In June 2020 a thread about *yangxifu* had 44,457 followers and 393,310 posts on a popular Chinese social media platform called Tieba.^3^ Another thread on ‘foreign wives’ on the same platform had 15,979 followers and 72,660 posts. People’s interest in *yangxifu* was further demonstrated through the virality of stories about Max Mei in 2018. Mei, who is married to a Ukrainian woman, launched a dating service aimed at matching ‘high quality’ Chinese men and Ukrainian women. According to the *South China Morning Post*, an article about this dating service was among the newspaper’s most widely read articles of the year. In this article, Mei explains that he had sensed a market opportunity when many of his 800,000 Weibo^4^ followers left envious comments in response to the pictures he posted of himself and his wife on Chinese social media, urging him to launch a match-making company (Toropov 2018).

The interest in foreign wives should be understood in the context of China’s marriage landscape which is characterized by its skewed sex ratio. Nearly four decades of one child-policy and enduring preference for sons have led to a relative lack of women of marriageable age (Driessen and Sier 2019), which has spurred Chinese men to look across borders to find brides (Barabantseva and Grillot 2019; Barabantseva 2021). China’s poorest farmers who lose out in Chinese marriage markets are reported to marry brides from countries such as Vietnam, Cambodia, Myanmar, Laos and even Pakistan, whereas China’s richest men are described as being enabled by their wealth to cross racial boundaries and marry white/*yang* women (Barabantseva and Grillot 2019). In all scenarios, the women are perceived as marrying *into* China. This notion is further strengthened by the workings of the patrilinear and patrilocal Chinese family systems, with a bride joining her husband’s family through marriage, and explains why western men who marry Chinese women are thought of as taking women away from China, while western women marrying Chinese men are considered ‘a gain for China’.

## Research methods

This article focuses on online materials about relationships between Chinese men and white western women. During the winter of 2019–2020 I selected six English-language websites that publish blogs written by women from western countries who are in relationships with Chinese men. Most of these websites were individual projects with occasional guest bloggers, and one website is a crowd-sourced platform with dozens of very relevant entries. This website is **c**alled WWAM-BAM, an acronym for Western Women & Asian Men, Breaking all Moulds. It has published WWAM content for several years, ranging from reviews of movies featuring WWAM couples to an agony aunt category and regular personal blogs discussing life in China for women in relationships with Chinese men. The stated mission of this website is helping women in such relationship with navigating cultural differences while also confronting stereotypes about ‘Asian men and culture’.^5^ Many of the contributors to this website are between 25 and 40 years old, have migrated to China on their own accord, and are now in stable romantic relationships with or married to Chinese men. Their entries target other white western women with a similar migration and relationship status, yet, as shown by this website’s comments section, the website also attracts women in WWAM relationships outside China and Chinese men who are interested in dating white/*yang* women. The Chinese men who comment on the discussions started by the women on this platform write in English and have often lived in English-speaking countries.

Studying these online environments offers an interesting opportunity for studying how racial knowledge is produced, contested, and reconsidered in the context of racialized relationships. The contents of the blogs are often highly reflexive and the frequent posting, in combination with people being able to comment on one another’s blogs, leads to a conversational atmosphere. The mixed audience brings different histories and languages of racialization into one conversation, made possible by the shared understanding of the audience and contributors’ conceptual worlds. Many of the women writers speak Chinese fluently and are familiar with racial concepts in Chinese, while the Chinese commenters are also familiar with western debates about race and racism. On an abstract level, these blogs can thus be understood as a sense-making project involving multiple histories of racialization. These websites are therefore an ideal site for a bottom-up study into the production of racial knowledge in the intimate realm of romantic, racialized relationships. I focus on terms that cross the boundaries between two worlds, such as the term *yang*, that is frequently used by both the western women writers and the Chinese commenters. Additionally, to understand how the term *yang* is used in Chinese-language discussions about romantic relationships between white women and Chinese men, I have analysed discussions in the Chinese cybersphere about this type of relationship, on the platforms Zhihu and Tieba – which are comparable to the English-language platforms Quora and Reddit.

I analysed the content of these websites and forums using a thematic analysis approach. First, I analysed forty blogs and two hundred social media posts to tease out the most important themes. I focused on blogs and posts that discussed race-related topics. After considering this material on a semantic level, I analysed the collected data on a latent level, which means that I identified how underlying assumptions, beliefs and ideologies shaped these writings (Braun and Clarke 2006, 84). I used only writings that were publicly available on websites that did not require a login or community membership.^6^ My knowledge of Mandarin and English enabled me to analyse the content in both languages and my long-term experience of living in China brought my attention to the underrepresentation of couples made up of white/*yang* women and Chinese men in the literature on racialized and transnational relationships.

## The conflictual characterisation of a y*angxifu*

In China, the word combination ‘white woman’ is not generally used to refer to white-looking women. Instead, such women are often referred to with the gender-neutral term *laowai*, which translates to ‘foreigner’ (Liu and Self 2020). However, foreign women who are married to Chinese men are also called *yangxifu*, meaning *yang-*wife. According to the *Pleco* dictionary, the term *yang* means ‘foreign (especially western)’, yet it is broadly understood as referring to white women married to Chinese men. In this section, I explore what being *yang* means and how women experience becoming *yangxifu.*

Ed Pulford’s analysis of *yang*’*s* various social and linguistic meanings over the past century and a half shows that *yang* has long indicated a sense of future orientation, following from projected ideas about which parts of the world were most advanced on an imagined spectrum of development (Pulford 2016, 477). *Yang* thus indicates foreignness, but only in a very particular manner, as it refers only to the parts outside China that are emulated for being ‘more developed’. In today’s China, the fact that the term *yang* still carries positive connotations is illustrated by the popularity of the term for promoting overseas products such as *yangjiu* (foreign liquor), in the positive connotations of the term *yangqi* (foreign flavour), which is often used as meaning ‘fashionable’ or ‘stylish’, and in the status derived from being called a *yangxifu* (foreign wife).

In their blogs, women often embrace the term *yangxifu*, which shows that they have acquired an understanding of local frameworks of race-making, but they also comment on the double-sidedness of the experience of becoming a *yangxifu*. One the one hand, they are admired, as y*angxifu* are considered beautiful and as enhancing their husbands’ status. In the blogs written by white western women, they write that their whiteness makes them their husband’s ‘ultimate bling’. One woman writes that having a *yangxifu* is ‘like cruising a BMW’, as it can make a man’s social status soar. Yet there is also attention for the other side of this coin, as women describe how their *yang* status leads to them feeling objectified, sexualized and not taken seriously. For example, in her blog about life as a white/*yang* woman in China, Jane writes:

Foreign women fail at being virtuous and pure, fail at being long-suffering self-sacrificing upholders of family and tradition. Foreign women date around before marriage, have sex with whomever they feel like (and enjoy it), they drink, they smoke, and they don’t obey their husbands or their in-laws. Almost any foreign woman who has been in China for any length of time will have come across the stereotypical attitude from certain people – from the guys at the club who assume you’re theirs for the asking, to the co-worker who asks if foreign women all sleep around after marriage, to the guy who asks you out and wonders out loud if he’ll be able to satisfy your voracious sexual appetite, to your Chinese boyfriend’s parents, who tell him that you can play with foreign girls, but they aren’t marriage material. It is these sorts of attitudes that are the truly infuriating part of being a female expat in China, not the problems with Western men or the lack of datable guys, but how you are looked upon.(Blog by Jane, in English, 5 May 2010)

In this quote, Jane explains that the perceived lack of romantic options for white/*yang* women in China results from ideas about *yang*-ness, instead of white foreign men’s lack of interest in them. She explains that even if dating a white/*yang* woman is considered as status enhancing for Chinese men, based on the perception of these women as being well-positioned in global hierarchies of race and development, her *yang-*ness also evokes suspicion. White/*yang* women can be thought of as less suitable for family life or downright promiscuous and are sometimes confronted with stereotypes pertaining to their sexuality. Another example of *yang* being used in a negative way can be found in the term *yangguizi*, or ‘foreign bastards’, which was used to refer to colonial soldiers, who were not only considered as possibly good business partners, but also as bearers of danger and violence (Pulford 2016, 476).

### Is she yang or is she white?

To what extent do the terms white and *yang* overlap and where do they differ? Even though *yang*-ness does not explicitly refer to a white skin colour, discussions about *yangxifu* make it clear that this term is solely reserved for women who are phenotypically white. In popular usage it can therefore be difficult to identify the differences between these two terms. *Yang*-ness is often understood in combination with nationality, which means that *yangxifu* is not a homogenous category. The most prominent division within this category is between the western *yangxifu* (which includes women from all countries seen as ‘western’) and the Russian *yangxifu* (which includes women from Russia and East-European countries). The main difference between these two types of *yangxifu* is their perceived positionality in a global hierarchy of nation-states. Western *yangxifu* are thought of as coming from relatively wealthy societies and being without urgent material needs. Therefore, their getting romantically involved with Chinese men is often described in terms of sacrifice and ‘true love’. They are seen as women who choose their partner without making material calculations. At the same time, their independent status raises doubt about their suitability as wives and daughters-in-law. Russian *yangxifu* are not considered in such romantic terms, but are portrayed as women who use transnational marriages with wealthy Chinese men as a vehicle for upward social mobility, leading to online warnings about Russian women being ‘all about the money’.^7^ At the same time, these women are considered as better suited for performing traditional gender roles in the Chinese family and ‘importing Russian brides’ is promoted at the state-level as a strategy for dealing with the fall-out from the relative low number of Chinese women of marriageable age. This is illustrated by an advertisement with the text ‘Russian wife + Chinese husband = ideal couple’ that circulated in Russian print media soon after Xi Jinping visited Moscow in May 2015 (Barabantseva and Grillot 2019).^8^

Russian and Western *yangxifu* have their whiteness in common, which is a factor that separates them from marriage migrants to China from countries such as Vietnam, Cambodia, Myanmar, Laos and even Pakistan, who are not considered *yang* and are often described as being ‘kidnapped’ or as being victims of human trafficking practices (Hodal 2017). As illustrated by the post below, written on Tieba, a woman’s white skin has the power to divorce her reputation from the status of her country. This means that white women from a ‘developing country’, such as Ukraine, can still be ‘a symbol of beauty and nobility’, while a woman from an Asian country that is ranked lower in the hierarchy of global power, such as Vietnam, can never compensate for the low status of her country, no matter what her individual status may be: Vietnam simply cannot compare with Ukraine!

According to the traditional Chinese meaning: the term overseas people (*yangren)* refers to white people in western countries such as Europe, America, and so on. Therefore, the term *yangxifu* also refers to women from these countries who marry Chinese men. We can’t call Vietnamese people *yangren*. In the eyes of Chinese people, Vietnamese people are lower than Chinese people. Even if they are rich, we can’t see Vietnamese people that way. But Ukrainians, even if they are from a developing country, they are still white people, and they are still what we call *yangren*, it does not matter what their income is. Even if conditions in China are better than in Ukraine, we still consider Ukrainian women as symbolizing beauty and nobility. Chinese men would be proud to marry them, but they would never be proud of marrying a Vietnamese woman (unless they really can’t find a wife within China). (Written by ‘no foreign wife, no marriage’, 25 May 2009, translated from Chinese by the author)^9^

This post illustrates how the terms whiteness and *yang*-ness are conflated and shows that even if *yangxifu* is not a homogeneous category in the Chinese context, the shared whiteness of women from different backgrounds within this category connects them by being associated with beauty and nobility. Research on the history of whiteness in China shows that these associations are based on the fact that fair skin has been considered as a symbol of refinement in China since antiquity and the racial categories and hierarchies of global power introduced to China with the arrival of the Europeans (Johansson 1998). According to Johansson, a fair skin tone first distinguished the Chinese elite from lower class workers in China and later worked to construct difference between Chinese and foreign women. However, even though whiteness, and particularly the whiteness of foreign women, has long been admired, it has also been associated with loose morals and promiscuity, casting a tinge of suspicion on the *yangxifu* (Johansson 1998).

In this article, I focus on the writings of *yangxifu* from western countries whose *yang-*ness is read as being strongly positioned in both hierarchies of race and class.

### He must be rich!

In their blogs, women frequently mention being confronted with the class dimension communicated through their *yang*-ness. Linda, a Dutch woman in her thirties who blogs about being married to Zhao, a Beijinger, explains that every time she and her husband take a taxi in Beijing, the driver assumes that Zhao is her guide or translator. When the couple subsequently explains that they are in fact husband and wife ‘their eyes go wide, and they tell my husband: “Oooh … then you must have a lot of money!”’ These reactions reveal that only wealthy Chinese men are perceived as being able to make a good match for white/*yang* women, illustrating the elevated status associated with these women’s *yang-*ness.

The imaginings underpinning these ideas are in line with what Constable (2003) refers to as ‘global hypergamy’, which is an imagined pattern in transnational romance that sees marriage as a vehicle for female upward social mobility. Following the logic that women need their husbands to provide for them, women are thought to pay close attention to a man’s economic and social status, and aim to ‘marry up’, into a more developed region or country, a better race, or a higher social class. Empirical research into transnational marriages has shown that the real-life stories of couples hardly ever resemble such imaginings, as there is no simple up or down, and transnational relationships, like all others, are complex, driving many contradictory processes of mobility (Constable 2005). However, in China, where a relative lack of women of marriageable age has only intensified the idea that men need to outdo – or at least match – women in terms of their status and economic success if they want to marry, these ideas continue to hold sway.

Interestingly, the Chinese men who the women writers on the WWAM-websites date and marry do not generally belong to China’s richest groups. The partners of these women include a musician, a PhD student, a human relations professional, a market salesman and a hydrogeologist. It is not uncommon for women in these relationships to earn higher salaries than their husbands or even become the family breadwinner. Linda reflects on the effects of the income difference between herself and her husband in a blog post that was commented on by 37 people. Linda first came to China in 2005 as a language student and later developed her professional career in Beijing working in projects ran by the EU and the Dutch government. While she was living and working in Beijing, she earned the income of a Dutch civil servant, which amounted to approximately twice the average salary earned in Beijing’s public sector and exceeded what her husband earned as a musician. In her blog, Linda writes that her husband suffers from this situation and refuses to join in activities that require spending money, such as going to cafes or taking trips, if he cannot contribute to the cost. She writes:

I feel bad for him feeling this way, because I don’t see his financial situation as a problem. I fell in love with him because of the man he is, not because I thought that one day cash would come flowing in because of his profession and I wouldn’t have to worry about money anymore.

(Blog by Linda, in English, 16 January 2015)

Linda’s blog gave rise to a lively discussion between Chinese men and women in relationships with Chinese men, who sometimes indicated they were in a similar position to Linda. The advice that was shared by the commenters focused on Linda’s husband needing to increase his earnings and Linda needing to adjust her behaviour (go down to his spending level, pool your money) to alleviate the situation. The commenters relied on culturalist views that men in a ‘traditional oriental culture’ cannot be confident in a relationship with a woman who is the breadwinner. According to one comment, these attitudes have developed ‘over thousands of years of evolution’ and are therefore difficult to change, making a relationship such as Linda’s unsustainable. None of the commenters critiqued the importance of the patriarchal family structures that give rise to the idea that husbands need to earn more than their wives to feel confident and happy in a relationship. The *yang-*factor only intensifies these dynamics as Chinese men in relationships with white/*yang* women not only need to fulfil the gendered expectations placed on the male provider but are also expected to compensate for the high status associated with their wives’ *yang-*ness.

## When racial worlds meet: Asian males in China

Racial meaning-making in an international and bilingual relationship is a two-way street. Even though the couples in this article are based in China and often communicate in Chinese, the racial language dominant in the women’s home country shapes the way they write about their relationships. The English-language blogs about WWAM relationships make use of racial labels popularized in the U. S. A. One clear example of this is the concept of the ‘Asian male’, which is prevalent on blogs about WWAM relationships – a term that itself incorporates the ‘Asian male’ category, illustrating the dominance of North American or western racial language and debates in these blogs. For example, in the U.S.A the undesirability of Asian men in the US dating landscape has been frequently discussed in scholarship and the media. Scholars confirm that statistically, Asian men are relatively unsuccessful in dating markets and argue that this should be understood in relation to the depiction of Asian men in North American popular culture either as asexual, shy, and effeminate, or as buffoonish and goofy (Eng 2001; Kao, Stamper Balistreri, and Joyner 2018). Kumiko Nemoto has even argued that dating white women is a strategy employed by Asian-American men to ascend the masculinity hierarchy in the U.S.A (2008). These discussions also shape the content produced by white, western women in relationships with Chinese men in China. For example, on the group website about WWAM romance in China there is a special section entitled ‘Where’s Wang?’ that is dedicated to critiquing the absence and negative depiction of Asian men in US popular culture.

### Why the surprise?

Why are women so surprised to find love in China? This is a question that several women discuss in their blogs about their relationships. They admit that before moving to China they could not imagine dating a Chinese man and thought about their time in China as a period without love and romance. After falling in love with a Chinese man, they look back and question their initial attitudes. Jacqueline, an American woman married to a Chinese man, remembers how she had secretly declared her year of teaching English in China to be ‘a lonely one without a single chance of dating’. After her love life in China turned out very differently from how she had imagined it, she wondered why she had assumed that she would remain single in China:

Was it merely that I grew up in an incredibly white middle-class suburb (I could count on one hand the Asian men I knew from kindergarten to high school graduation)? Was it the overwhelming absence of positive images of Asian men in the whitewashed world of American popular culture? (Blog by Jacqueline, in English, 24 August 2015).

Riana, another American woman married to a Chinese man, asks herself similar questions when she remembers her scepticism towards the idea of dating in China: “Looking back, I’m not sure why I found the thought of finding love in China so humorous and inconceivable. In a country of 1.3 billion people, the majority of them male, why did finding a boyfriend seem so implausible?” (Blog by Riana, in English, 5 July 2013). She self-critically concludes that it might have been her own arrogance and closed-mindedness that nearly stood between her and finding the love of her life.

The feelings of surprise that marked the start of Jacqueline and Riana’s relationships are widely shared among women in WWAM relationships, which implies that racial ideas about ‘Asian men being less desirable’ influence the way they think about Chinese men in China. Unlike racialized relationships between people who share a nationality, those involved in international and bilingual racialized relationships not only learn to look at their own society from a different racial perspective, as described in the literature about racial literacy, but also navigate an interaction between two histories of racialization in which they, sometimes unintentionally, reproduce the racial languages and logics of their home society. In the last section of this article, I focus on strategies for confronting negative reactions to their relationships that are discussed by white/*yang* women in the blogs about their relationships.

## Love as anti-racist activism

Blog posts on WWAM websites regularly address the issue of how to cope with being confronted with racial prejudices evoked by being in a racialized relationship. My research shows that white women in WWAM relationships often promote their own relationship as a form of anti-racism. The blogs about confronting racism show that these couples are subjected to various forms of racial abuse, ranging from the women being heckled on white supremacy websites for being ‘trash’ and ‘traitors’, as Jacqueline describes, to being on the receiving end of stares and daily comments that are either based on negative ideas about Chinese men or on the surprise evoked by seeing Chinese men partnering with white/*yang* women. Considering these reactions, walking the streets of China’s cities as a WWAM couple or publishing content about one’s WWAM relationship can therefore be seen as a bold anti-racist activity, as Jacqueline explains:

A lot of people still think interracial love is wrong. There was a time when I used to think blogging about interracial love was just about promoting diversity and understanding. But now I think it’s so much more – it’s about combating hatred too. So if you’re blogging about interracial love, just consider that every post you publish is a bold statement in support of interracial couples everywhere. Let’s support love, together. (Blog by Jacqueline, in English, 15 December 2016)

Lea, who lives with her Chinese husband in a small Chinese city, wrote to a WWAM-advice platform with questions about how to deal with being stared at whenever she and her husband are out in public. She explains: ‘It’s like locals can’t believe that a foreign woman and a Chinese guy could be together’. In response to her questions, the agony aunt encourages Lea not to let stares stop her from showing affection and instead think of showing her relationship as ‘community service’. Her advice is as follows:

So yeah, there’s no way to stop this entirely because you will always meet new people, both Chinese and foreign, that will think your relationship is ‘weird’ or bad or whatever. But don’t let it stop you. Proudly put pics of you and your guy on WeChat and Facebook. Hold hands in public. Go out and be seen. It’s tiring dealing with the comments and stares but don’t stop being affectionate in public or being proud of your guy.

You can’t stop other people from doing it, but you can change the way you see it. See it as a little community service. You’re educating people and maybe they’ll go home and tell their friends what they saw and then it will become more acceptable. Even if people just see you holding hands, that’s enough to be memorable and talked about. We WWAM-ers need to pay it forward a little and hopefully through our openness about it, it will help everyone become more used to western women with Asian men.^10^ (Response by agony aunt, in English, 5 July 2017)

In addition to resisting negative responses to WWAM relationships through normalizing these relationships by showing public affection and celebrating WWAM romance on social media platforms, some white*/yang* women go one step further either by declaring that they love their Chinese partners *for* their race or arguing that negative stereotypes about Asian or Chinese men do not apply to *all* these men. I conceptualize statements in these realms as belonging to the ‘loving orientalist’ and ‘rare gem’ narrative, which I argue tend to reproduce the racial language it aims to attack.

A blog written by Lisa provides a typical example of the loving orientalist narrative, as it shows that Lisa subscribes to the idea – albeit in the most loving way – that a Chinese race exists and that her husband is part of it. Moreover, she states that this is an important reason for loving him. She writes:

I love all these traits that make him; I simply cannot begin to understand why he would ever wish them gone. If he did by some fairytale or horror story-type transformation become a stereotypical privileged white dude, I would probably not be interested in him anymore. And it’s not about the looks; it’s about the culture and the character attached to his race. He is the man I love, he is who he is, humble, helpful and hard-working, because of his race and because of his culture. (Blog by Lisa, in English, 13 May 2017)

This blog invites reactions, particularly from men who live in the U.S.A and self-identify as Asian, such as Ed:

As a traditional Asian man, I am always very happy and proud to be Asian although I don’t look down on other races. Asian culture has solid working ethic and morality. We have the intelligence and the best food. Asian also have the highest IQ of all races. (Response by Ed, in English, 13 February 2018)

These accounts reproduce ideas about race by putting out positive narratives about men whom they conceptualize as belonging to a Chinese or Asian race. Even if this approach is effective in countering negative stereotypes about Chinese or Asian men, it also threatens to strengthen the belief that people can be categorized into several races that are defined by a set of characteristics inherent to these categories. The characteristics highlighted in these accounts are reminiscent of orientalizing ideas that demarcate the boundaries between ‘East’ and ‘West’ and put forward essentialising ideas about what it means to belong to either world (Said 1978).

Women who produce the rare gem narrative do not challenge negative racial stereotypes but argue that those stereotypes are not applicable to *all* Chinese men. In their blogs, as exemplified by this reaction to a blog about Zhang’s (2009) research on Western women’s (negative) perceptions of Chinese men, love declarations go hand in hand with the reproduction of negative ideas about Chinese men:

This article is inter[e]sting. My husband doesn’t spit, drink, smoke, gamble or go out daily or have long fingernails; I was surprised about the spitting to be a problem. I only see my students and elderly folks do that. He’s a clean guy, though he does love to tell me a story from his college days when he went a month without bathing! I didn’t realize that these shortcomings mentioned in the article were a big reason for the lack of western women with Chinese guys.

However, I must say I’ve never met another guy who I would consider marrying due to their love of smoking, drinking and going out (among other things). I always tell my husband he’s a rare gem. (Response by Charlene, in English, 25 April 2010)

This rare gem narrative is a recurrent theme that implies that Chinese men who are married to white, western women are exceptional for not adhering to the racial stereotypes projected onto *other* Chinese men and shows how romantic relationships across racial boundaries do not necessarily contribute to breaking down racial categories. On the contrary, by claiming that their husbands are exceptions to the rule these women reinforce stereotypes about Chinese men.

## Conclusion

China is no longer only a place from which many migrants depart; it also has become a popular destination for migrants around the world, giving rise to new patterns of global intimacy. As scholars have previously argued, relationships between people ascribed to different racial identities may be a micro site where racial meanings are negotiated, produced, and reproduced (Twine and Steinbugler 2006). This article has analysed the writings of white/*yang* women about their romantic relationships with Chinese men (whom they sometimes conceptualize as ‘Asian’) on WWAM websites. It has shown that women who write on these websites are united in their desire to break down the gendered and racial moulds that render their relationships exceptional yet produce narratives about their relationships that both challenge and reproduce racializing discourses.

Whereas most scholarship on racialized romance has focused on relationships between people ascribed to different racial categories in multi-ethnic societies (Fernandez 2010; Osuji 2013; Sue 2013; Steyn, McEwen, and Tsekwa 2019), this article has analysed relationships that do not only cross racial but also linguistic and national boundaries. This means that the people in these relationships are not only racialized differently, but they also express themselves through racial language rooted in different histories of racialization. By bringing this case to the literature on racialized romance, this article has connected debates about the production of racial knowledge in the intimate realm with scholarship on multiple languages of racialization. In doing so, it has shown that emerging patterns of migration, such as increased migration from western countries to China, drive the renegotiation of racial identities and hierarchies. This work is part of a growing body of literature that views China as a key site for the development of new racial understandings following the country’s rapid rise as a global powerhouse.

## Notes

1. In this article, I use the term ‘racialized’ instead of ‘interracial relationships’ to reflect a social constructivist approach to racial categories while also acknowledging that race is usually the first attribute to be noticed about relationships between people who are ascribed to different racial categories (Steyn, McEwen, and Tsekwa 2019, 1682).

2. This is the term used by Jiehai Zhang.

3. Tieba is a popular Chinese social media website where people discuss all sorts of questions.

4. Weibo is comparable to Twitter.

5. On this website, the words ‘Asian’ and ‘Chinese’ are used interchangeably. Yet in this online environment ‘Asian’ should be understood as a placeholder for ‘Chinese’.

6. This research was approved by the ethical board of the University of Amsterdam. I use pseudonyms where authors used their real names and only provide links for online content that does not reveal authors’ identities.

7. For example, in the comments to this YouTube video about Ukrainian women marrying Chinese men: https://www.youtube.com/watch?v=o081wqsny7w&t=7s.

8. Article published in *Beijing News* promoting the ‘importing of brides’, 22 January 2015: https://cutt.ly/jujNYAw.

9. Link to the thread on Tieba where this comment was posted: https://tieba.baidu.com/p/582577306.

10. The link to the anonymous advice column: https://www.wwambam.com/dear-ms-wai-deal-haters/.
